# Transcription variants of SLA-7, a swine non classical MHC class I gene

**DOI:** 10.1186/1753-6561-5-S4-S10

**Published:** 2011-06-03

**Authors:** Rui Hu, Gaëtan Lemonnier, Emmanuelle Bourneuf, Silvia Vincent-Naulleau, Claire Rogel-Gaillard

**Affiliations:** 1 INRA, UMR de Génétique Animale et Biologie Intégrative, Jouy-en-Josas, France; 2 CEA, DSV, iRCM, Laboratoire de Radiobiologie et Etude du Génome, Jouy-en-Josas, France; 3 AgroParisTech, UMR de Génétique Animale et Biologie Intégrative, Jouy-en-Josas, France

## Abstract

In pig, very little information is available on the non classical class I (Ib) genes of the Major Histocompatibility Complex (MHC) i.e. *SLA-6*, *-7* and *-8*. Our aim was to focus on the transcription pattern of the *SLA-7* gene. RT-PCR experiments were carried out with *SLA-7* specific primers targeting either the full coding sequence (CDS) from exon 1 to the 3 prime untranslated region (3UTR) or a partial CDS from exon 4 to the 3UTR. We show that the *SLA-7* gene expresses a full length transcript not yet identified that refines annotation of the gene with eight exons instead of seven as initially described from the existing RefSeq RNA. These two RNAs encode molecules that differ in cytoplasmic tail length. In this study, another *SLA-7* transcript variant was characterized, which encodes a protein with a shorter alpha 3 domain, as a consequence of a splicing site within exon 4. Surprisingly, a cryptic non canonical GA-AG splicing site is used to generate this transcript variant. An additional *SLA-7* variant was also identified in the 3UTR with a splicing site occurring 31 nucleotides downstream to the stop codon. In conclusion, the pig *SLA-7* MHC class Ib gene presents a complex transcription pattern with two transcripts encoding various molecules and transcripts that do not alter the CDS and may be subject to post-transcriptional regulation.

## Background

The Major Histocompatibility Complex (MHC) class I gene family comprises classical (Ia) and non classical (Ib) genes. The highly polymorphic class Ia genes are widely expressed and encode membrane-bound glycoproteins that present self and viral peptides to cytotoxic T cells [1] and modulate the activity of natural killer cells [2]. In contrast, the class Ib genes display limited polymorphism, and are predominantly expressed in immunotolerant organ sites in human, notably at the feto-maternal interface [3]. In man, three MHC class Ib genes have been characterized, namely *HLA-E*, -*F* and *-G*[3] and *HLA-G* has been shown to express alternatively spliced variants encoding various membrane-bound as well as soluble proteins [4]. In mouse, the *H2-QaI* gene is orthologous to *HLA-E*[5] and functional homologies have been established between *H2-Qa2* and *HLA-G*[6]. One to four MHC class Ib genes have been identified in rat according to haplotypes [7] and four MHC class Ib genes have been characterized in cattle [8]. There is a growing interest in addressing the role of the MHC class Ib genes in the species where they are characterized. Indeed, MHC class Ia genes seem to share similar functions across species but the MHC class Ib genes are good candidates to address questions on both shared and species-specific immunity-related roles.

In pig, very limited information is available on the MHC class Ib genes *SLA-6*, *-7* and *-8*. The three genes have been fully sequenced from the homozygous Hp1a.0 haplotype [9,10]. Nine allelic variants have been reported for *SLA-6* and only two for *SLA-7* or *SLA-8*[11]. It has been shown that *SLA-Ib* genes are expressed in a less restricted manner than the *HLA-Ib* genes [12,13] despite a predominant transcription in the lymphoid organs, the lung and the digestive tract [13]. In addition, conversely to the *SLA-Ia* genes, transfection experiments have revealed that the promoters of *SLA-7* and *SLA-6* do not respond to interferon, suggesting distinct regulatory systems for pig MHC class Ia and Ib genes, as in human [14]. Our aim was to focus on the transcription of the *SLA-7* gene known to have a unique reference transcript [12]. In this report, we show that the *SLA-7* gene expresses a full-length transcript not yet identified as well as at least two additional alternative spliced variants that lead to either exon alteration in the resulting protein or modification of the 3’end of the transcript.

## Methods

### Animals, tissues and RNA extraction

Tissues from Melanoma-bearing Libechov Minipigs (MeLiM) [15] and French Large White pigs were used. The tissues from MeLiM pigs have been sampled on 13 months old animals. At the time of tissue sampling, all MeLiM animals had regressed, meaning that they were not bearing melanomas anymore [15]. Tissues included brain, thymus, tonsil, spleen and liver. Total RNA was extracted using QIAGEN RNeasy Mini Kits (Qiagen, France). All RNA samples were purified by on-column digestion of DNA with DNase I as recommended by the manufacturer (Qiagen, France).

### Primer design

**Figure 1:**
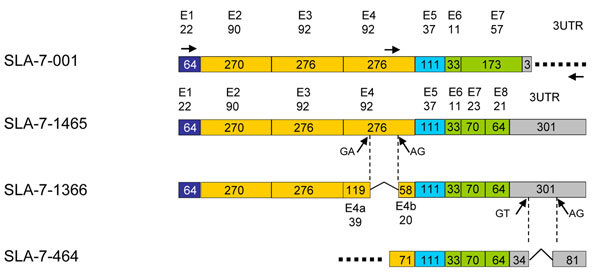
**Schematic representation of the SLA-7 transcripts.** Exons are numbered E1 to E8 and the three prime non coding region is indicated as 3UTR. Sizes of exons and 3UTR are given in nucleotides within the boxes. The number of aminoacids for each exon is indicated above the exon number. Exons represented by a dark blue box (E1) correspond to the leader sequences. Exons represented by orange boxes (E2 to E4) stand for the alpha1, 2 and 3 domains of the molecules. Exons represented in bright blue (E5) correspond to the transmembrane domain. Exons represented by green boxes encode the cytoplasmic tail of the molecule. The 3UTR is represented by a grey box. Positions of the primers used for RT-PCRs are indicated by arrows on top of the figure on E1 (SLA-7-e1-F), E4 (SLA-7-e4-F) and 3UTR (SLA-7-3UTR-R). The donor and acceptor splice sequences are positioned by arrows on E4 and 3UTR boxes.

Three primers were designed from the *SLA-7* reference cDNA [12] and genomic [9,10] sequences, using the Primer3 online program [16]. The primer combinations were suitable to amplify the full coding sequence from exon 1 to the three prime untranslated region (3UTR) or a partial coding sequence from exon 4 to the 3UTR (Table 1 and Figure 1). Primers were also designed to amplify cDNAs of the *RPL32* gene that has been used as control gene for expression levels (Table 1 and Figure 1).

**Table 1:** RT-PCR primers

Genes	Primer name	Sequence of primers (5’-3’)	Position of primers	Accession numbers
SLA-7	SLA-7-e1-F	ATGGGGCCCCGAGCCCTCCTCCT	Exon1	
	SLA-7-e4-F	TGGAGAGGAGCAGAGCTACA	Exon4	AJ251914
	SLA-7-3UTR-R	AGAGCCACTGCTGATCCAGT	3’UTR	AY463541

RPL32	RPL32-F	TGCTCTCAGACCCCTTGTGAAG	Exon1	
	RPL32-R	TTTCCGCCAGTTCCGCTTA	Exon2	NM_001001636

### RT-PCR and sequencing

Two micrograms of DNaseI-treated total RNA were reverse-transcribed (Superscript II enzyme, Invitrogen, USA) with Oligo (dT) primers in a final volume of 20 µL to which 30 µL of water were further added to prepare the stock solution of RT samples. PCRs were carried out in a final volume of 15 µL using 100 nM of each primer, 1 µL of the 1:10 RT sample and the GoTaq™ DNA polymerase (Promega, USA). Thermocycling conditions were as follows: 94°C for 3 min, followed by 35 amplification cycles at 94°C for 30 sec, 60°C for 30 sec and 72°C for 90 sec, followed by a final extension at 72°C for 5 min. The PCR products were purified using the JETQUICK Gel Extraction Spin Kit (Genomed, Germany) for further cloning into pCR2.1 vector (TA Cloning Kit, Invitrogen, USA) and sequencing (Eurofins MWG Operon, France).

### Sequence analysis

Sequence similarities were searched with the BLAST tools [17]. Multiple alignments were carried out with CLUSTALW [18]. cDNA sequences were translated to protein by online DNA to Protein translation tool (http://bio.lundberg.gu.se/edu/translat.html).

## Results and discussion

### SLA-7 full coding sequences

**Figure 2:**
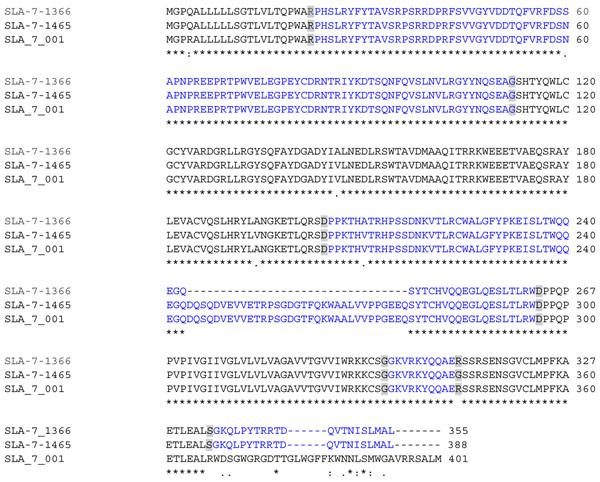
**Multi-alignment of peptides encoded by the transcripts SLA-7-1465, SLA-7-1366 and SLA-7-001**. The successive eight (SLA-7-1366 and SLA-7-1465) or seven (SLA-7-001) exons are alternatively indicated by black and blue font. Aminoacids at the junction between two exons are in grey boxes. Aminoacid similarities between two or three sequences are indicated below the sequence alignments by dots or stars, respectively.

Full length *SLA-7* transcripts were characterized by RT-PCR from the thymus of MeLiM pigs using the primers SLA-7-e1-F and SLA-7-3UTR-R (Table 1 and Figure 1). A 1465 nucleotides long transcript was obtained and further named SLA-7-1465 (Accession number: GU322918). Annotation was carried out by aligning the cDNA sequence to the genomic reference sequence (GenBank accession number AJ251914) and eight exons were detected in this new transcript, in contrast to the reference full-length transcript (Accession number NM_213768) that harbours only seven exons [12] (Figure 1) and is referred to as SLA-7-001 (OTTSUST00000000782) in the Vertebrate Genome Annotation database [19]. The two RNAs encode proteins that differ in the cytoplasmic tail (Figure 2). The SLA-7-001 encoded protein contains a cytoplasmic tail that is defined by exons 6 and 7 and is 68 aminoacids long. The SLA-7-1465 encoded protein is characterized by a cytoplasmic tail that is defined by exons 6 to 8 and is 55 aminoacids long. It has been demonstrated that the cytoplasmic tail of MHC class I molecules contributes to their expression on the cell surface [20] and that mutations of cysteine residues in the cytoplasmic tail of MHC class Ia molecules modify extracellular recognition by Leukocyte Ig-Like receptor 1 [21]. Moreover, it has been reported that HLA-F molecules are entirely dependent on the cytoplasmic tail for export from the endoplasmic reticulum to the Golgi apparatus [22]. Altogether, these reports strongly support a major role for the cytoplasmic tail of MHC class I molecules in transport and function. Further experiments are required to study whether the SLA-7 molecules encoded by SLA-7-001 or SLA-7-1465 transcripts have distinct properties due to their different cytoplasmic tails.

### An SLA-7 spliced variant encoding a protein with a shorter alpha 3 domain

A 1366 nucleotides long transcript was retrieved from brain RNA and further referred to as SLA-7-1366 (accession number: HQ224544**)**. Surprisingly, annotation of the cDNA revealed the presence of nine exons due to a splicing site within exon 4 (Figures 1 and 2). The two exons matching to exon 4 were named exons 4a and 4b (Figure 1). Alignment of the SLA-7-1366 cDNA to the reference genomic sequence showed that between exons 4a and 4b, the donor and acceptor splice sites were GA and AG, respectively. This finding indicates that a cryptic non canonical splicing code is used to express this *SLA-7* transcript variant. The general rule is the use of GT and AG for donor and acceptor splicing sites, respectively [23], but alternative codes may be functional [24]. It has been shown that the GA-AG splicing site is rarely used and a few cases have been reported among which splicing in the human *parafibromin* gene [25]. Our results suggest that the *SLA-7* gene may be subject to subtle regulation resulting in the use of rarely used non canonical splicing sites. Additional studies are required to analyze whether this regulation is tissue-specific.

The SLA-7-1366 and SLA-7-1465 encoded molecules with different alpha 3 domain lengths (figures 1 and 2) i.e. 59 (39 from exon 4a and 20 from exon 4b) and 92 aminoacids long, respectively. The alpha 3 domain corresponds to the Immunoglobulin-like region and interacts with the cell surface CD8 glycoproteins that are expressed on cytotoxic T lymphocytes and function as a co-receptor with the T cell receptor [26]. Expression of SLA-7 molecules on the cell surface has not been demonstrated. However, the alpha 3 domain encoded by the SLA-7-1366 transcript is shortened by comparison to the full-length molecule, suggesting that such a modification may alter interactions with cell receptors.

### A spliced variant in the 3’UTR with no alteration of the encoded protein

By using primers targeting the three prime end of the gene from exon 4 (SLA-7-e4-F) to the 3UTR (SLA-7-3UTR-R) (Table 1 and figure 1), two partial transcripts were recovered that differ in non coding sequence length (Figure 1). The 650 nucleotide long sequence (SLA-7-650) corresponds to the expected full-length sequence. The 464 nucleotide long sequence SLA-7-464 (accession number: GU322919) is the result of an alternative splicing within the 3UTR region, 31 nucleotides downstream to the stop codon. Modification of the 3UTR does not affect the encoded molecules. As indicated in figure 1, the canonical GT-AG rule was used for this splicing. A new category of transcripts has been recently characterized that can be subject to non sense mediated decay (NMD) [27,28]. Variants targeted by NMD can present alternative splicing in the 3UTR and the distance between the stop codon and the splice site has been shown to be more than 50 nucleotides long [29]. The SLA-7-464 variant cannot fall into this category of transcripts subject to NMD because the distance between the stop codon and the splicing site in only 31 nucleotides. However, it is tempting to hypothesize that SLA-7-464 variants are subject to a post-transcriptional regulation that has to be explored.

**Figure 3:**
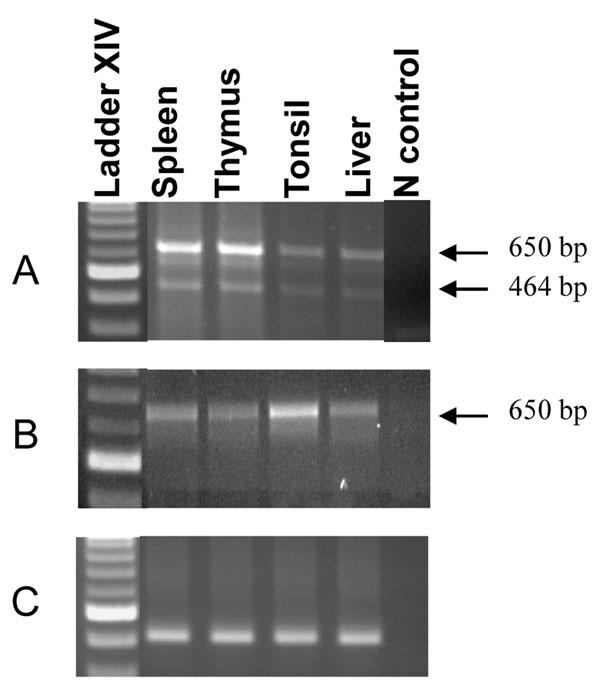
**Tissue expression patterns in MeLiM and Large White pigs**. Detection of the *SLA-7* transcript variants in adult tissues from MeLiM (A) and Large White (B) pigs by RT-PCR using the primers SLA-7-e4-F and SLA-7-3UTR-R. The *RPL32* gene was used as a control for expression levels as shown for four tissues of MeLiM pig (C).

The expression patterns of these two 3UTR variants were studied in four different tissues including spleen, thymus, tonsil, and liver from MeLiM and Large White pigs. Surprisingly, the SLA-7-650 band was detected in all tissues of both breeds but the SLA-7-464 band was detected only in MeLiM pigs (Figure 3). We cannot rule out a very weak expression of the short variant in Large White pigs but our results strongly suggest a co-expression of both variants in MeLiM pigs and a predominant expression of the SLA-7-650 variant in the Large White pigs included in our study.

## Conclusion and perspectives

We have identified an *SLA-7* full length transcript that had not been characterized before and that differs from the reference sequence by the length of the encoded cytoplasmic tail. In addition, we show that the *SLA-7* gene is subject to alternative splicing transcription that leads to either a transcript encoding a molecule with a shortened alpha 3 domain or a transcript that is spliced in the 3UTR after the stop codon. In conclusion, the non classical MHC class Ib gene *SLA-7* gene presents a complex transcription pattern, the regulation of which needs to be further investigated. The functions of the putative encoded molecules also need to be studied.

## List of abbreviations used

MHC : Major Histocompatibility Complex; HLA : Human Leukocyte Antigen; SLA : Swine Leukocyte Antigen; MeLiM : Melanoblastoma-bearing Libechov Minipigs; 3UTR : 3 prime untranslated region; Class Ib : non classical class I; Class Ia : classical class I; CDS: coding sequence; NMD : Non sense Mediated Decay; PCR : Polymerase Chain Reaction; RT : reverse transcription.

## Competing interests

The authors declare that they have no competing interests.

## Authors' contributions

RH participated in working out the experimental design, performed all experiments and sequence analysis, and drafted the manuscript. GL carried out RT-PCR experiments. EB and SVN contributed to tissue sampling. CRG coordinated the whole study, contributed to the experimental design, the analysis and interpretation of the results and corrected the manuscript. All authors have read and approved the final manuscript.
